# Supplementary material for: The PINK1—Parkin mitophagy signalling pathway is not functional in peripheral blood mononuclear cells
Source: PLoS One. 2021 Nov 11;16(11):e0259903. doi: 10.1371/journal.pone.0259903 (PMC8584748; doi:10.1371/journal.pone.0259903)
Supplement: S1 Fig — Jurkat cells were treated with 20 μM CCCP for increasing periods of time and the ubiquitination of MFN1/MFN2 was analysed by immunoblotting. The levels of ubiquitinated MFN1 and MFN2 were quantified relative to the respective non-ubiquitinated protein. Graph represents mean ± SEM; MFN1, n = 2; MFN2, n = 3. (PDF) [file pone.0259903.s001.pdf]

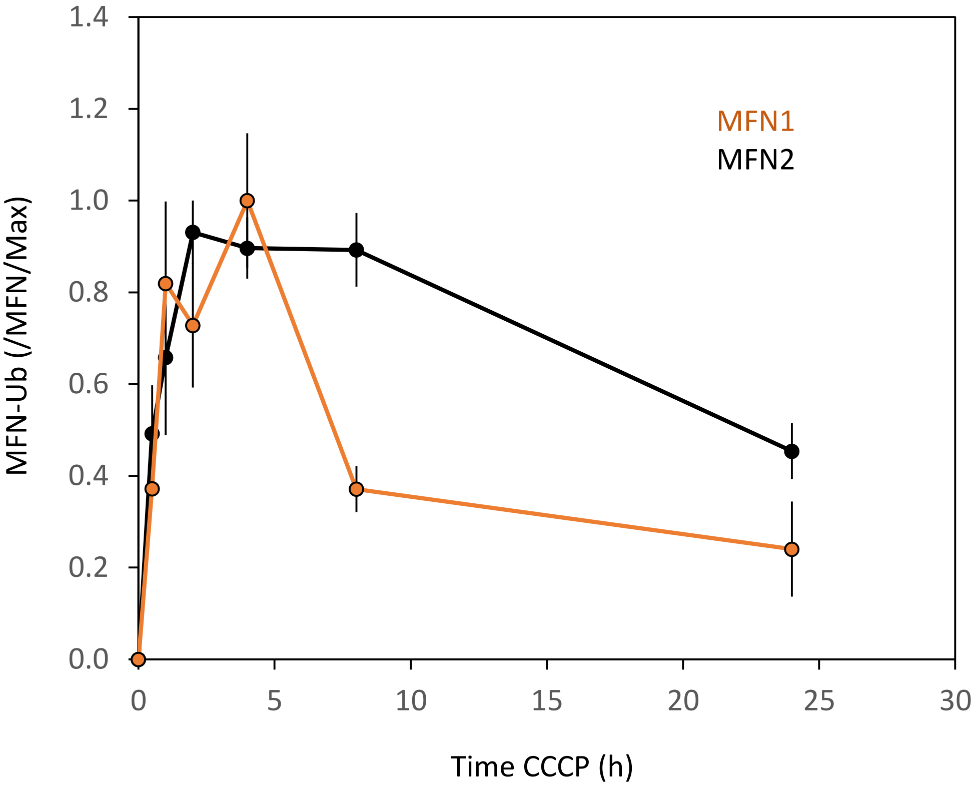

S1 Fig. Time course analysis of MFN ubiquitination in Jurkat cells. Jurkat cells were treated with 20 µM CCCP for increasing periods of time and the ubiquitination of MFN1/MFN2 was analysed by immunoblotting. The levels of ubiquitinated MFN1 and MFN2 were quantified relative to the respective non-ubiquitinated protein. Graph represents mean ± SEM; MFN1, n=2; MFN2, n=3.
