# Supplementary material for: The PINK1—Parkin mitophagy signalling pathway is not functional in peripheral blood mononuclear cells
Source: PLoS One. 2021 Nov 11;16(11):e0259903. doi: 10.1371/journal.pone.0259903 (PMC8584748; doi:10.1371/journal.pone.0259903)
Supplement: S2 Fig — SH-SY5Y, fibroblast, Jurkat and PBMC cultures were left untreated or treated with 20 μM CCCP for 24 hours. Samples were analysed by western blotting with the PINK1 antibody clone D8G3 (Cell Signaling Technology). PINK1 protein is indicated by the red box. (PDF) [file pone.0259903.s002.pdf]

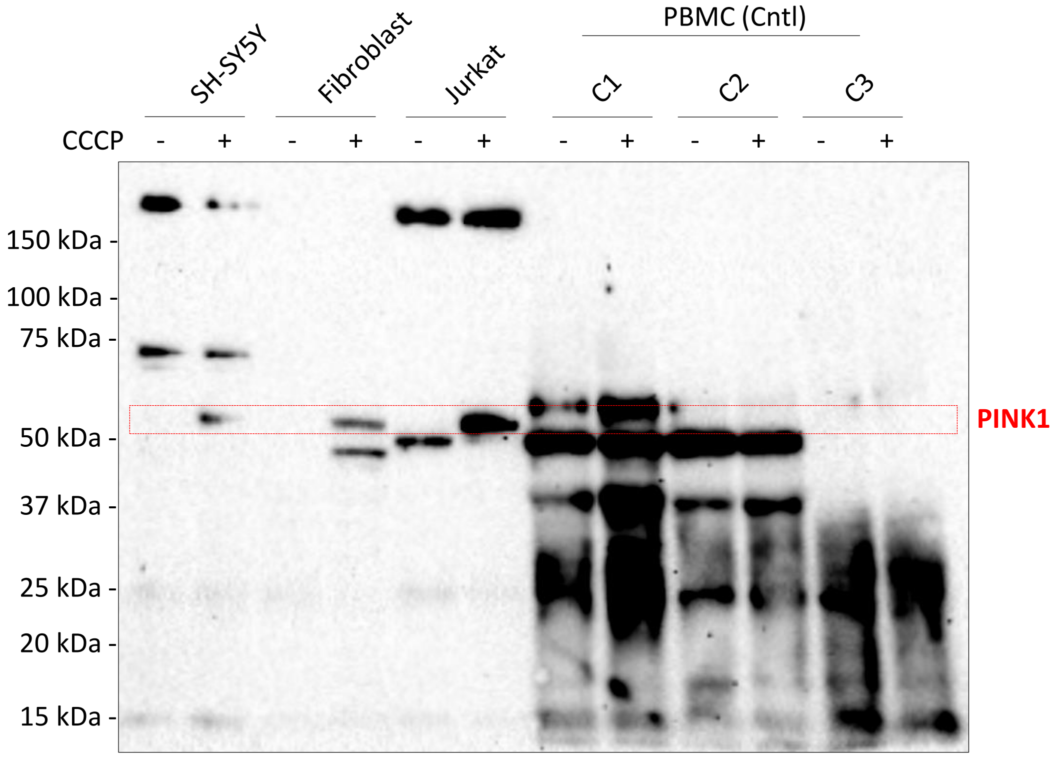

S2 Fig. Analysis of PINK1 expression in different cell types. SH-SY5Y, fibroblast, Jurkat and PBMC cultures were left untreated or treated with 20 µM CCCP for 24 hours. Samples were analysed by western blotting with the PINK1 antibody clone D8G3 (Cell Signaling Technology). PINK1 protein is indicated by the red box.
