# Supplementary material for: The PINK1—Parkin mitophagy signalling pathway is not functional in peripheral blood mononuclear cells
Source: PLoS One. 2021 Nov 11;16(11):e0259903. doi: 10.1371/journal.pone.0259903 (PMC8584748; doi:10.1371/journal.pone.0259903)
Supplement: S3 Fig — qPCR was performed on equal amounts of cDNA from each cell sample, and Ct values were expressed relative to the fibroblast (Fib) sample. (PDF) [file pone.0259903.s003.pdf]

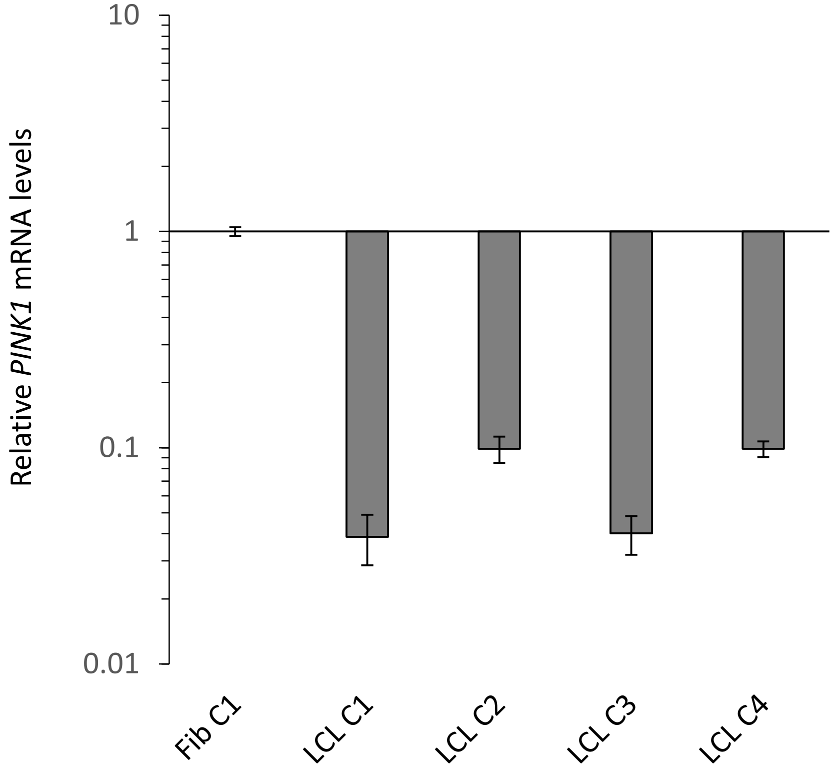

S3 Fig. Reverse transcriptase qPCR analysis of *PINK1* in LCLs. qPCR was performed on equal amounts of cDNA from each cell samples and C_t values were expressed relative to the fibroblast (Fib) sample.
