# Supplementary material for: The PINK1—Parkin mitophagy signalling pathway is not functional in peripheral blood mononuclear cells
Source: PLoS One. 2021 Nov 11;16(11):e0259903. doi: 10.1371/journal.pone.0259903 (PMC8584748; doi:10.1371/journal.pone.0259903)
Supplement: S1 Table — (PDF) [file pone.0259903.s004.pdf]

S1 Table. Age and sex of PMBC donors

PBMC donor	Age (y)	Sex
C1	31.2	Female
C2	26.4	Male
C3	25.3	Male
C4	28.6	Male
C5	60.1	Male
C6	64.2	Male
C7	66.9	Male
C8	74.2	Female
C9	27.7	Male
C10	58.8	Female
C11	31.4	Female
C12	50.4	Male
C13	34.1	Female
C14	58.8	Female
C15	72.8	Male
C16	64.6	Male
C17	67.5	Female
C18	63.1	Male
