# Supplementary material for: The PINK1—Parkin mitophagy signalling pathway is not functional in peripheral blood mononuclear cells
Source: PLoS One. 2021 Nov 11;16(11):e0259903. doi: 10.1371/journal.pone.0259903 (PMC8584748; doi:10.1371/journal.pone.0259903)
Supplement: S2 Table — (PDF) [file pone.0259903.s005.pdf]

**S2 Table. Characteristics of primary antibodies used in this study**

| Target | Host | Cat. No. (Supplier) | Immunogen | Clone | Application | Dilution |
|---|---|---|---|---|---|---|
| β-Actin | Ms[1] McAb | ab3280 (Abcam) | Chicken β-actin aa 50–70 | C4 | WB | 1:1000 |
| MFN1 | Ms McAb | ab57602 (Abcam) | Human Mitofusin 1 aa 1–741 | 3C9 | WB | 1:1000 |
| MFN2 | Ms McAb | ab56889 (Abcam) | Human Mitofusin aa 661–757 | 6A8 | WB | 1:1000 |
| Parkin | Rb McAb | 702785 (Thermo Fisher Scientific) | Human Parkin aa 1–465 | 21H24L9 | WB | 1:100 |
| Parkin | Ms McAb | 4211 (Cell Signaling Technologies) | Human recombinant Parkin | | WB | 1:500 |
| Parkin | Ms McAb | Sc-32282 (Santa Cruz Biotechnology) | Human Parkin aa 399–465 | | ICC | 1:50 |
| PINK1 | Rb PcAb | BC100-494 (Novus Biological) | Human PINK1 protein aa 175–250 | | WB | 1:1000 |
| PINK1 | Rb McAb | 6946 (Cell Signaling Technologies) | Residues surrounding aa 140 of human PINK1 | D8G3 | WB | 1:1000 |
| SDHA | Ms McAb | Ab14715 (Abcam) | Full length native SDHA | 2E3GC12FB2AE2 | WB | 1:1000 |
| TOMM20 | Rb PcAb | sc-11415 (Santa Cruz Biothechnology) | Full length human TOMM20 | | ICC | 1:100 |

[1]Abbreviations: aa, amino acid residues; ICC, immunocytochemistry; McAb, monoclonal antibody; Ms, mouse; PcAb, polyclonal antibody; Rb, rabbit; WB, western blot.
