# Supplementary figures and images for: The PINK1—Parkin mitophagy signalling pathway is not functional in peripheral blood mononuclear cells
Source: PLoS One. 2021 Nov 11;16(11):e0259903. doi: 10.1371/journal.pone.0259903 (PMC8584748; doi:10.1371/journal.pone.0259903)

Figure 1A

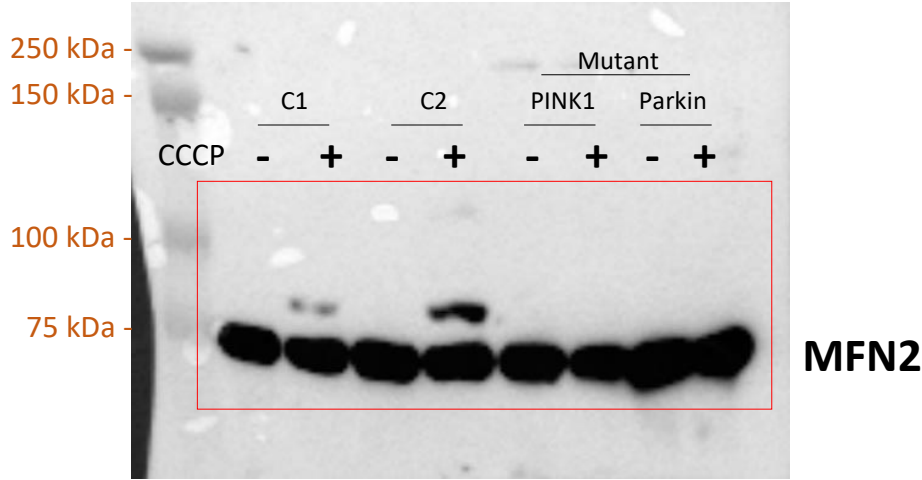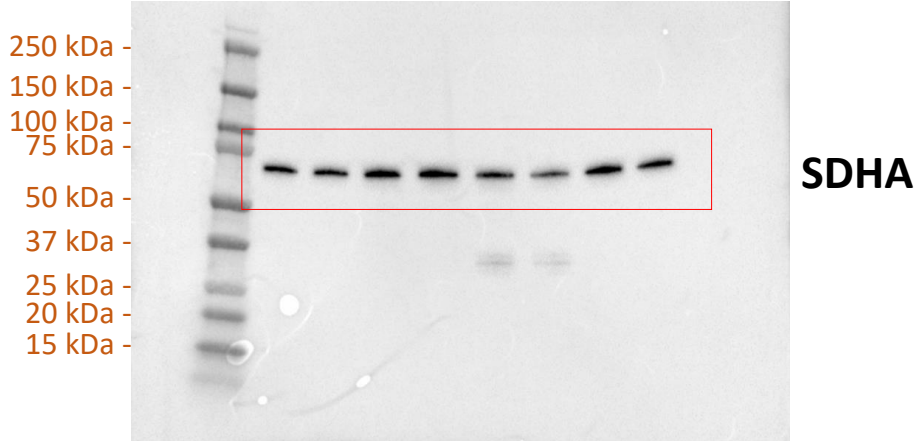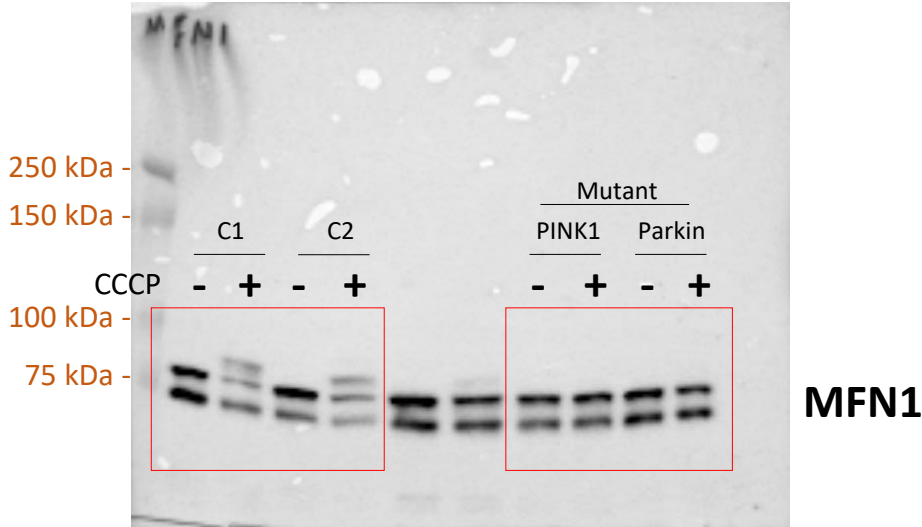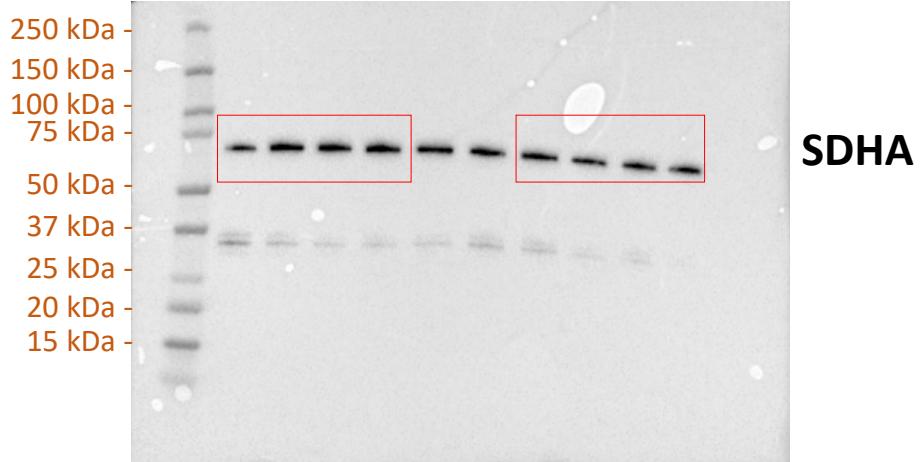

**Figure 2A**

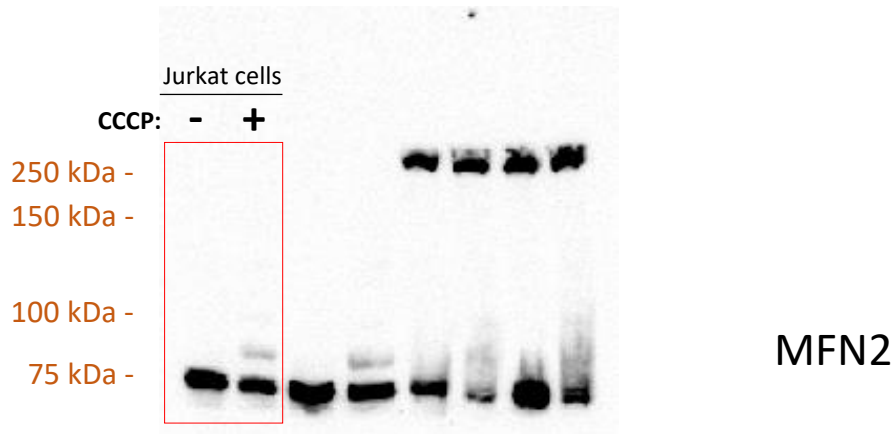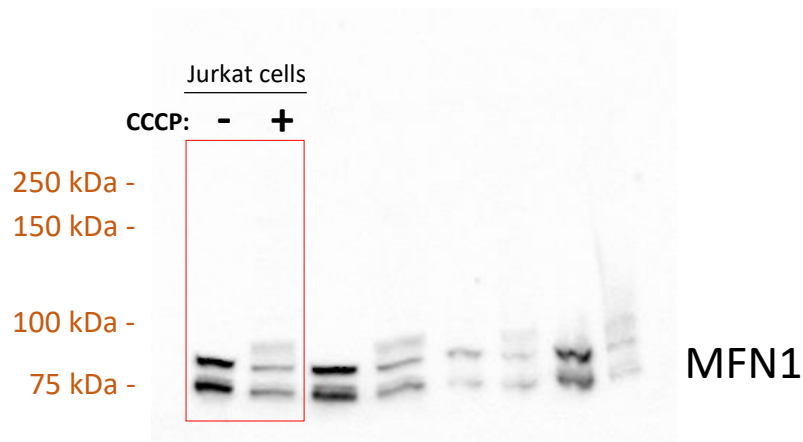

**Figure 2B**

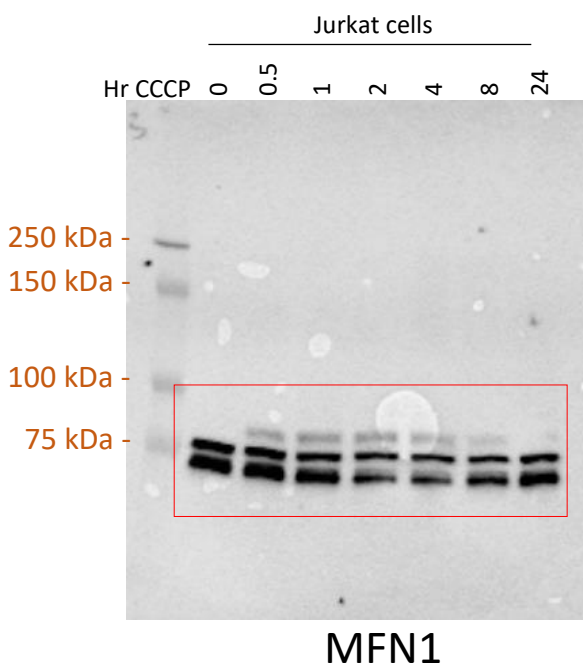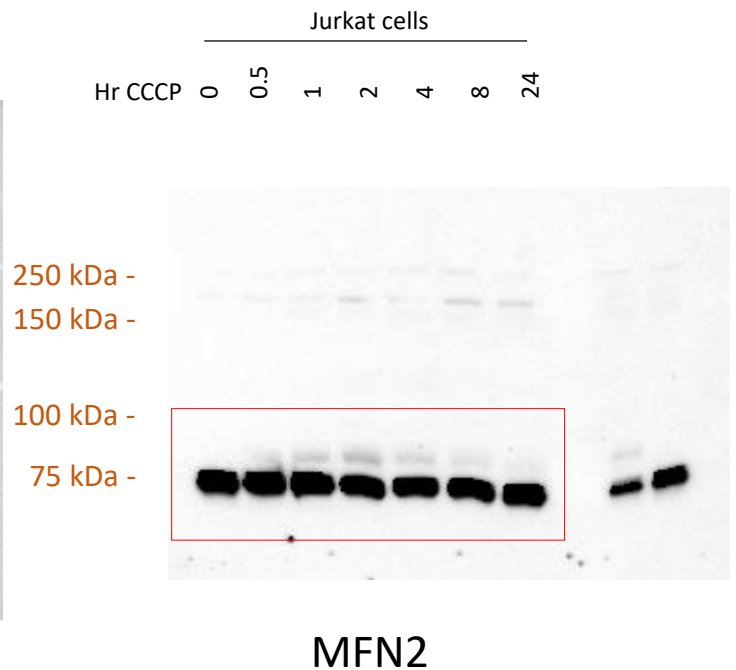

Figure 3

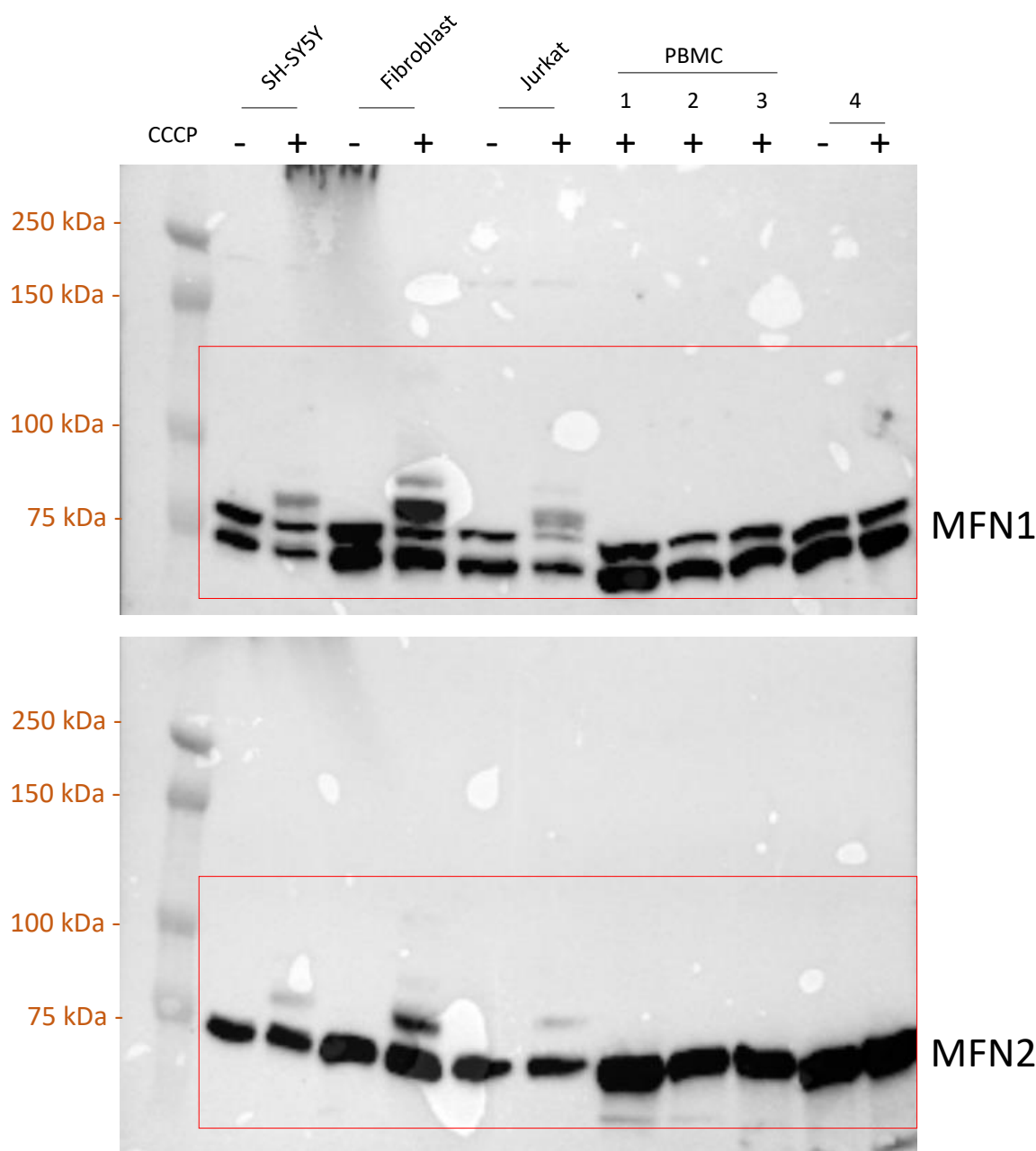

Figure 4

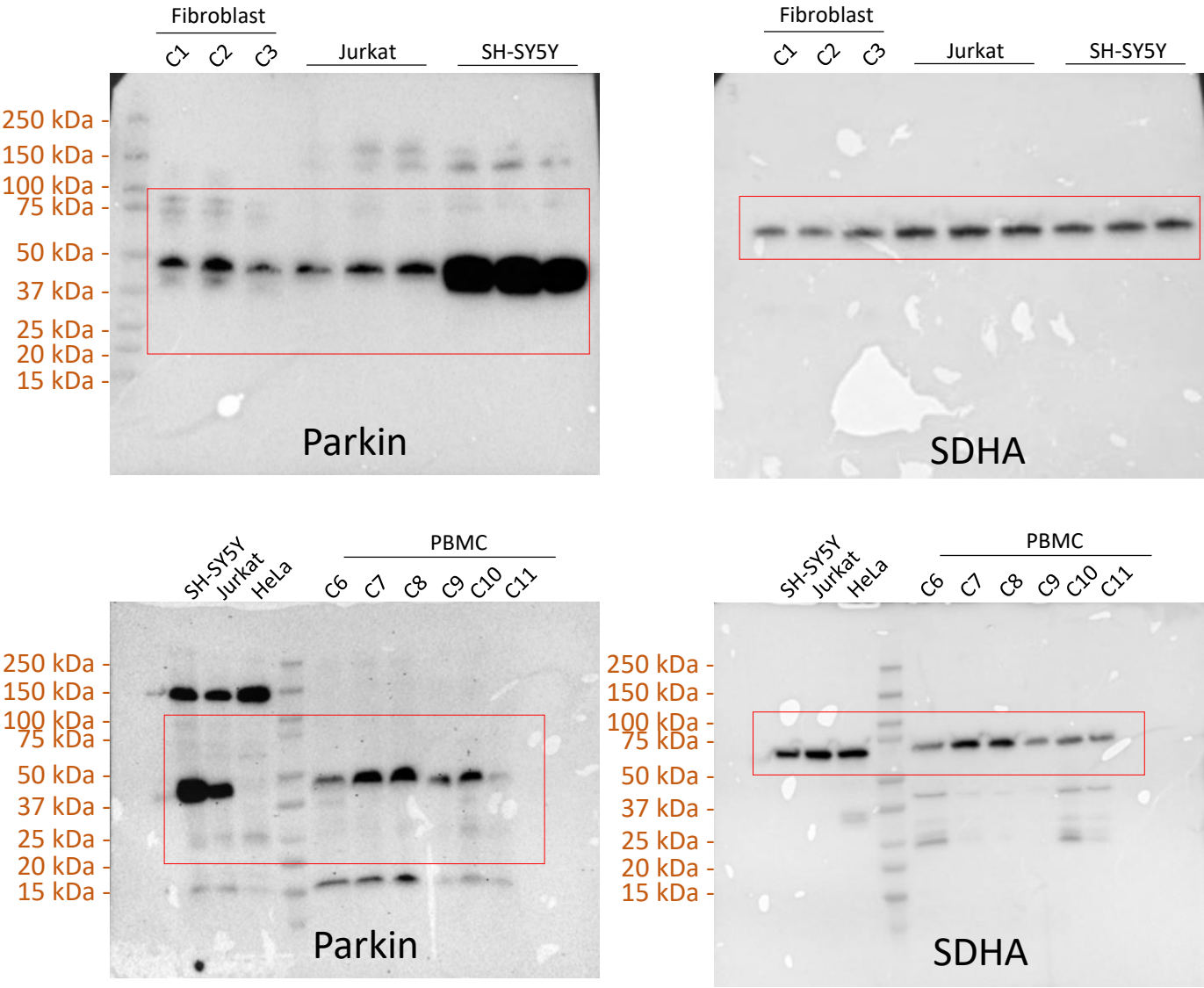

Figure 5

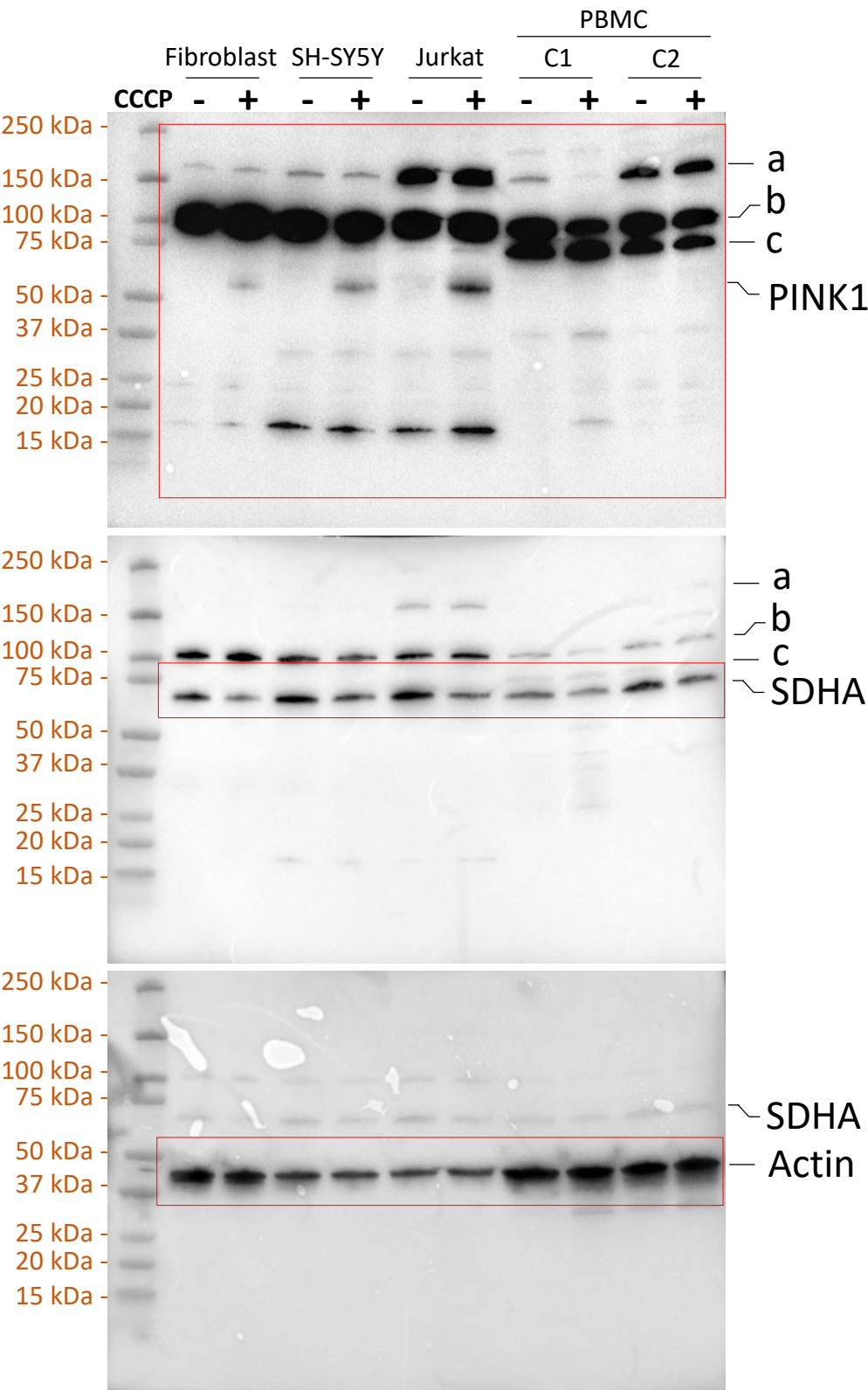

Figure 6B

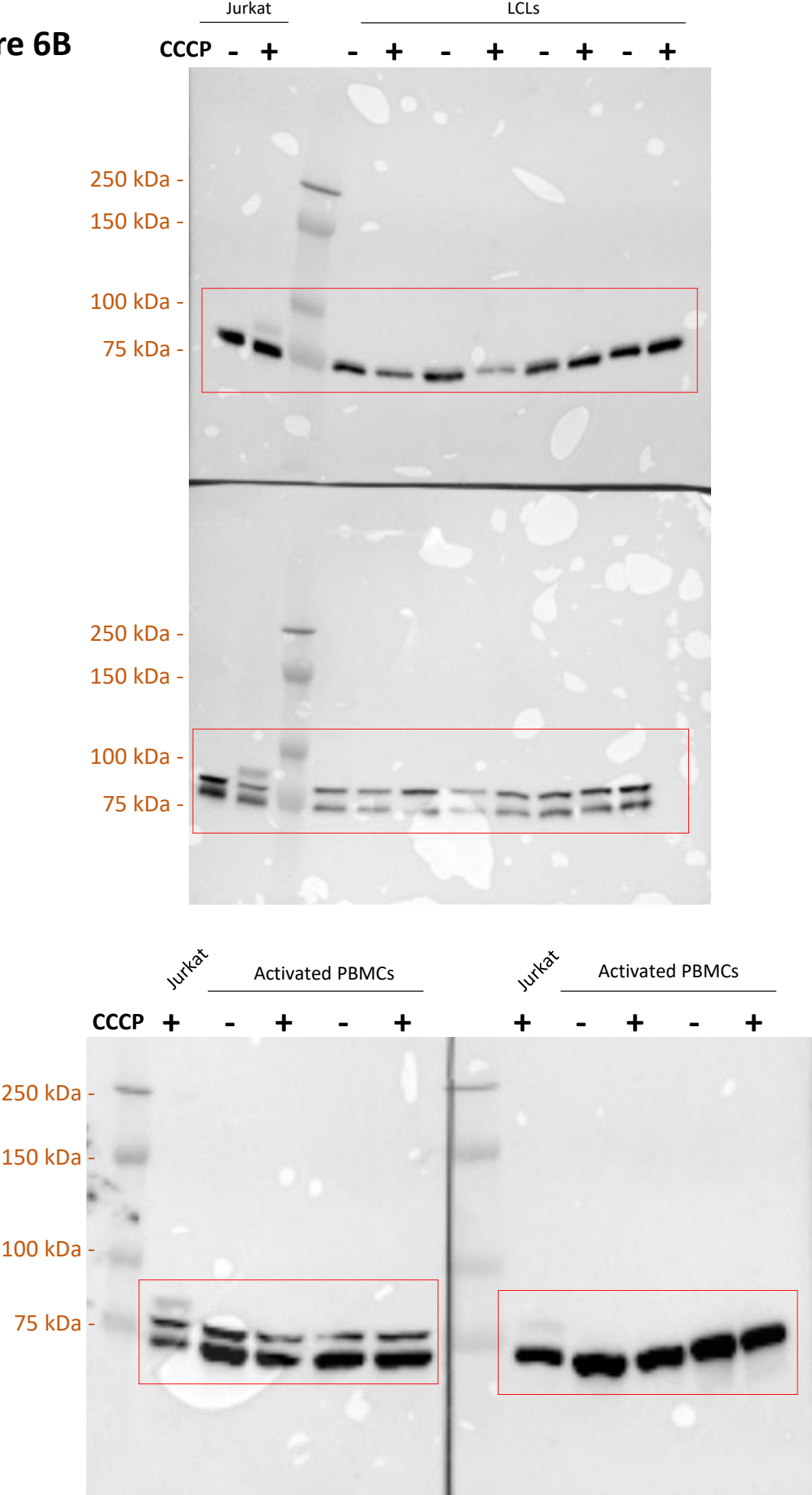

Figure 6C

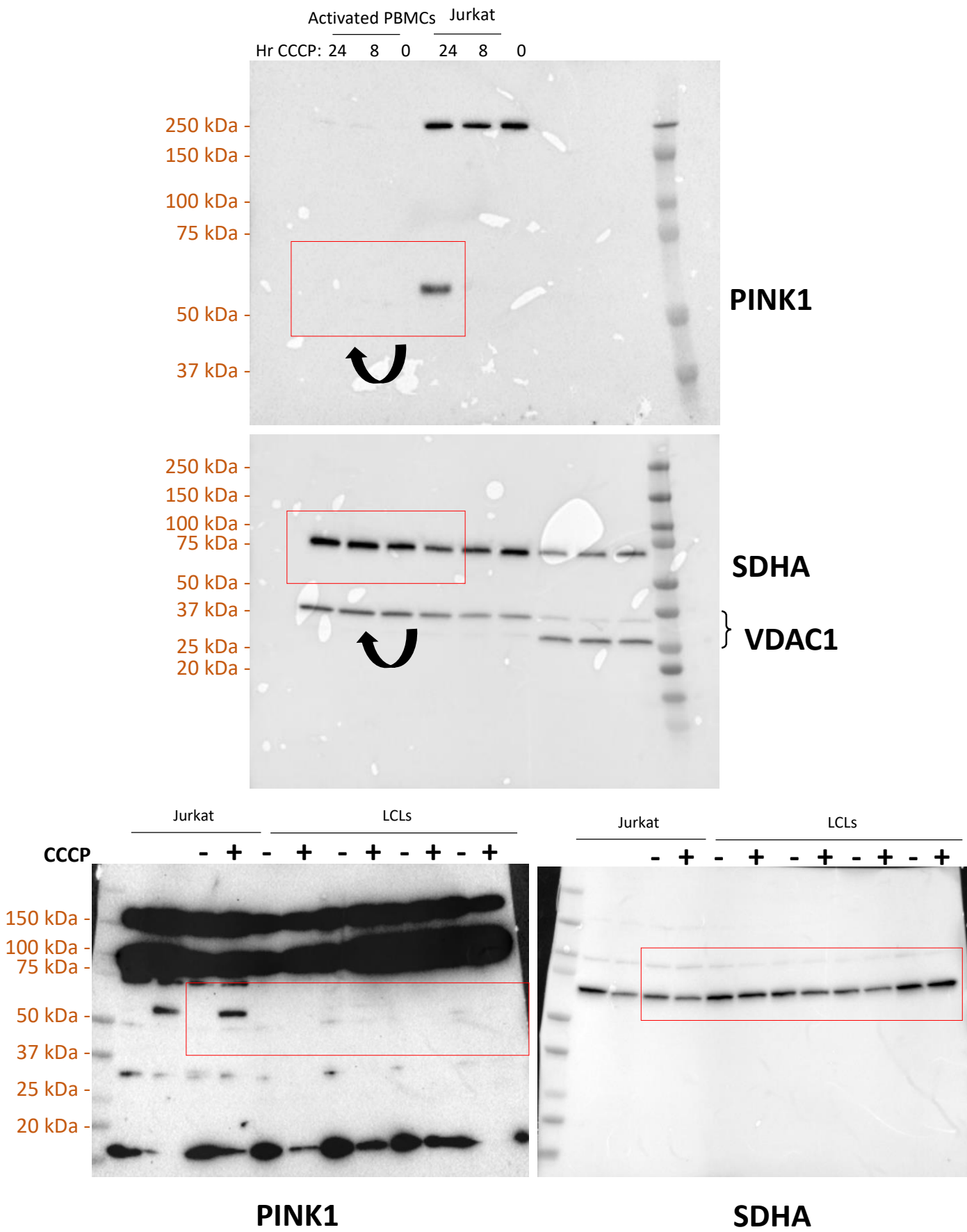

### Figure 6D

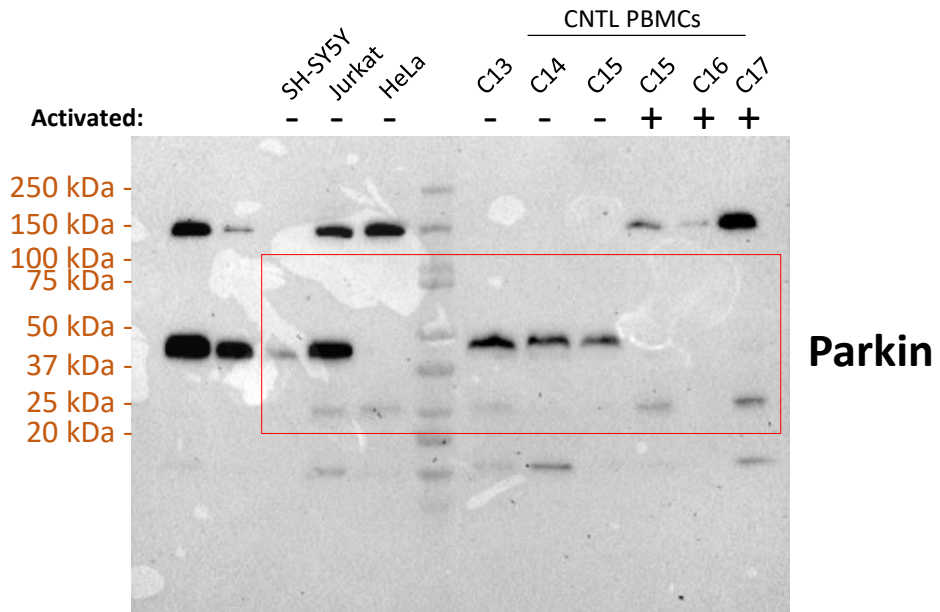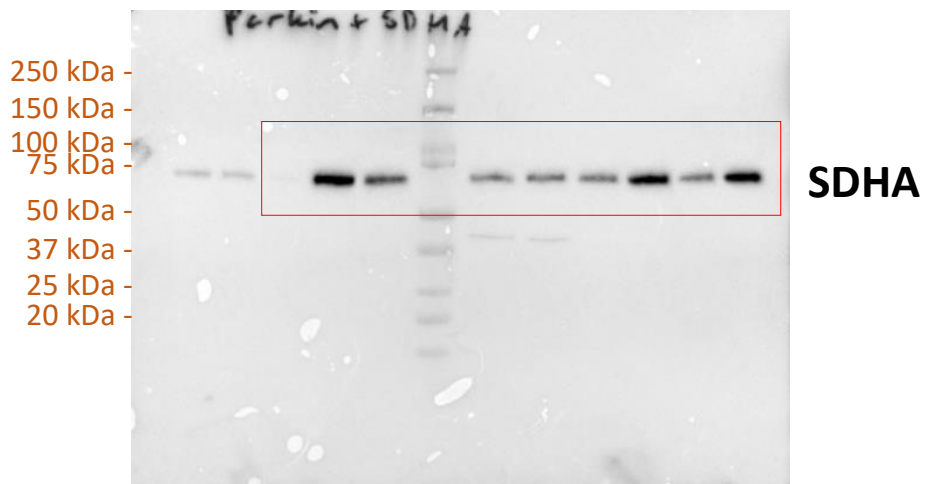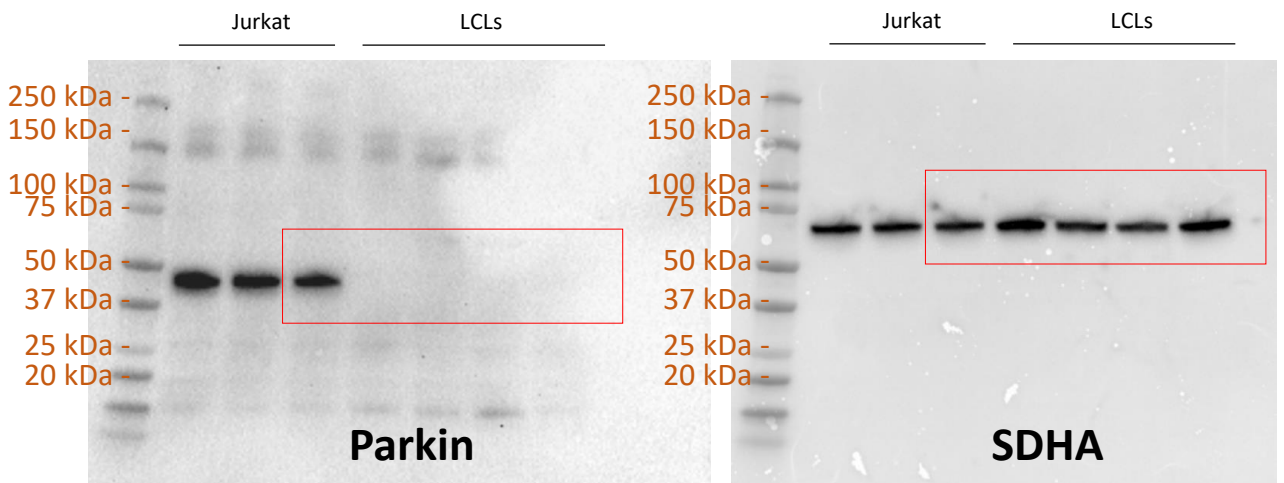

Supplement: S1 Raw images — (PDF) [file pone.0259903.s007.pdf]
