# Supplementary material for: The PINK1—Parkin mitophagy signalling pathway is not functional in peripheral blood mononuclear cells
Source: PLoS One. 2021 Nov 11;16(11):e0259903. doi: 10.1371/journal.pone.0259903 (PMC8584748; doi:10.1371/journal.pone.0259903)
Supplement: Attachment — Submitted filename: Response to reviewers.docx [file pone.0259903.s008.docx]

**REBUTTAL**

PONE-D-20-03884
The PINK1 – Parkin mitophagy signalling pathway is not functional in peripheral blood mononuclear cells
PLOS ONE

Dear Dr. Taanman,

Thank you for submitting your manuscript to PLOS ONE. After careful consideration, we feel that it has merit but does not fully meet PLOS ONE’s publication criteria as it currently stands. Therefore, we invite you to submit a revised version of the manuscript that addresses the points raised during the review process.

Please address all reviewer comments. In particular, it would be important to show that CCCP is inducing mitochondrial depolarization (R1) and assess the impact of CCCP treatment on Parkin distribution (R2).

**Response authors:** We have addressed all reviewer comments. In particular, we have now demonstrated that CCCP is inducing mitochondrial depolarization (new Fig. 4), and we have assessed the impact of CCCP on Parkin distribution in immunocytochemical experiments (new Fig. 7).

We would appreciate receiving your revised manuscript by Apr 12 2020 11:59PM. When you are ready to submit your revision, log on to <https://www.editorialmanager.com/pone/> and select the 'Submissions Needing Revision' folder to locate your manuscript file.

**Response authors:** Due to COVID-19 lockdown we were unable to resubmit manuscript by Apr 12 2020 11:59PM

If you would like to make changes to your financial disclosure, please include your updated statement in your cover letter.

To enhance the reproducibility of your results, we recommend that if applicable you deposit your laboratory protocols in protocols.io, where a protocol can be assigned its own identifier (DOI) such that it can be cited independently in the future. For instructions see: [http://journals.plos.org/plosone/s/submission-guidelines#loc-laboratory-protocols](https://eur01.safelinks.protection.outlook.com/?url=http%3A%2F%2Fjournals.plos.org%2Fplosone%2Fs%2Fsubmission-guidelines%23loc-laboratory-protocols&data=02%7C01%7C%7C8290d351010d429a8e1a08d7bbdc6aa7%7C1faf88fea9984c5b93c9210a11d9a5c2%7C0%7C0%7C637184427667948984&sdata=msIaGfdUJp7h835LleHwVf5ecyO4VW%2BGAvEbPCxY5cQ%3D&reserved=0)

Please include the following items when submitting your revised manuscript:

- A rebuttal letter that responds to each point raised by the academic editor and reviewer(s). This letter should be uploaded as separate file and labeled 'Response to Reviewers'.
- A marked-up copy of your manuscript that highlights changes made to the original version. This file should be uploaded as separate file and labeled 'Revised Manuscript with Track Changes'.
- An unmarked version of your revised paper without tracked changes. This file should be uploaded as separate file and labeled 'Manuscript'.

Please note while forming your response, if your article is accepted, you may have the opportunity to make the peer review history publicly available. The record will include editor decision letters (with reviews) and your responses to reviewer comments. If eligible, we will contact you to opt in or out.

We look forward to receiving your revised manuscript.

Kind regards,

Ivan R. Nabi, Ph.D.
Academic Editor
PLOS ONE
Reviewers' comments:

Reviewer's Responses to Questions

**Comments to the Author**

1. Is the manuscript technically sound, and do the data support the conclusions?

The manuscript must describe a technically sound piece of scientific research with data that supports the conclusions. Experiments must have been conducted rigorously, with appropriate controls, replication, and sample sizes. The conclusions must be drawn appropriately based on the data presented.

Reviewer #1: Partly

Reviewer #2: Yes

2. Has the statistical analysis been performed appropriately and rigorously?

Reviewer #1: Yes

Reviewer #2: Yes
3. Have the authors made all data underlying the findings in their manuscript fully available?

The [PLOS Data policy](https://eur01.safelinks.protection.outlook.com/?url=http%3A%2F%2Fwww.plosone.org%2Fstatic%2Fpolicies.action%23sharing&data=02%7C01%7C%7C8290d351010d429a8e1a08d7bbdc6aa7%7C1faf88fea9984c5b93c9210a11d9a5c2%7C0%7C0%7C637184427667958977&sdata=jMXAJnubczVYOuFaolWrO%2FNhxt5lxY6O1dsmmNC6G5s%3D&reserved=0) requires authors to make all data underlying the findings described in their manuscript fully available without restriction, with rare exception (please refer to the Data Availability Statement in the manuscript PDF file). The data should be provided as part of the manuscript or its supporting information, or deposited to a public repository. For example, in addition to summary statistics, the data points behind means, medians and variance measures should be available. If there are restrictions on publicly sharing data—e.g. participant privacy or use of data from a third party—those must be specified.

Reviewer #1: Yes

Reviewer #2: Yes
4. Is the manuscript presented in an intelligible fashion and written in standard English?

PLOS ONE does not copyedit accepted manuscripts, so the language in submitted articles must be clear, correct, and unambiguous. Any typographical or grammatical errors should be corrected at revision, so please note any specific errors here.

Reviewer #1: Yes

Reviewer #2: Yes
5. Review Comments to the Author

Please use the space provided to explain your answers to the questions above. You may also include additional comments for the author, including concerns about dual publication, research ethics, or publication ethics. (Please upload your review as an attachment if it exceeds 20,000 characters)

Reviewer #1: The submitted manuscript by Taanman et al. looks at the utility of PBMCs in order to functionally assay the activity of the PINK1/Parkin pathway as a potential diagnostic for Parkinson Disease. Overall, they conclude that the PINK1/Parkin mitophagy pathway is not active in PBMCs, primarily due to low expression of PINK1, and thus PBMCs are not an ideal cell type in which to examine this pathway. While overall the data are good and seem to support the overall conclusion, I have one major issue and a few minor comments.

The primary issue I have, is that the authors need to show that the CCCP treatment they use here is sufficient to promote loss of membrane potential in the PBMCs, as not all cells respond the same way to CCCP. If the PBMCs are not losing membrane potential with the CCCP treatment being used, this would explain the lack of PINK1 protein and the lack of MFN1/2 ubiquitination. Although the mRNA levels for PINK1 are low, this does not mean anything on its own as mRNA levels don’t correlate to protein levels, even in their own data for PINK1.

**Response authors:** We agree with the reviewer that we need to demonstrate that our CCCP treatment is sufficient to promote loss of inner mitochondrial membrane potential in the PBMCs. We have now included new cytochemical experiments with live Jurkat and PBMCs, which demonstrate that our CCCP treatment is sufficient to induce mitochondrial depolariation in these cell types (new Fig. 4).

Minor comments:
I also have some concerns about the PBMC cultures that were analyzed. Ideally, the same PBMC cultures would be examined across all three readouts (MFN1/2 ubiquitination / Parkin expression / PINK1 expression). Is there a technical reason this could not be done?

For Fig 4, looking at the expression of Parkin, it was not clear why the qPCR and western blots were not done on the same PBMC cultures. Furthermore, these were not the same lines examined for MFN1/2 ubiquitination.

**Response authors:** We agree with the reviewer that, ideally, the PBMC cultures from the same donors should be examined across all three readouts. However, our recruitment protocol for blood donors for this study, approved by the local Ethics Committee, did not allow this. Donors could only be asked for a blood sample once. Thus, we were unable to obtain repetitive fresh blood samples for PBMC isolation from the same subjects over the course of the project.

For Fig 5, looking at expression of PINK1, why was the protein analysis only done for two PBMC cultures? Adding a few more lines would strengthen the conclusions. For example, for the Parkin protein expression six different lines were examined.

**Response authors:** In the revised manuscript Fig. 5 has become Fig 6. We agree with the reviewer that more PMBC samples would strengthen the conclusions. Three additional PMBC samples were analysed with a different PINK1 antibody in Supplementary Figure 2 (S2 Fig).

Authors need to add load controls for western blots in Fig 2, Fig 3, Fig 6B.

**Response authors:** We think that it is unnecessary to add loading controls for western blots in Fig 2, 3 and 6B (Fig. 6B has become Fig 8B in the revised manuscript), because the aim of the experiments was not to determine the amount of MFN or MFN-Ub relative to another protein, the aim was to determine if MFN-Ub was detectable in the samples after CCCP treatment. The MFN signal serves as control that protein has indeed been loaded in the wells.

Could the authors clarify how mRNA expression was normalized? Did they use expression of a housekeeping gene (e.g. GAPDH / beta-actin)?

**Response authors:** As we compared transcript levels in different cell types, we could not use transcript levels of household genes, such as *GAPDH* or *ACTB*, for normalisation, because household genes are transcribed at variable levels in different cell types. Therefore, we normalised C_t_ values to cDNA quantity after accurate Qubit fluorometric quantitation. This explanation has now been added to the manuscript.

For the T-cell activation experiment, were any controls performed to show that T-cells were indeed activated?

**Response authors:** The cell proliferation of the T-cells after treatment with CD3/CD28 Dynabeads demonstrated that they were indeed activated. Untreated PBMC fractions do not show cell proliferation under our culture conditions.

Reviewer #2: The authors sought to determine if PBMCs would be useful cells to look for markers of the pink-parkin mitophagy pathway as possible future biomarker analysis in PD. Using MFN1/2 ubiquitination, pink and parkin expression analysis they conclude that this pathway is not active in this cell type. While there are several other important aspects of this pathway that could have been interrogated, the fact that Pink protein is undetectable would point to the downstream aspects being deficient. Since parkin is expressed, it might have been informative to interrogate the localization of parkin in PBMCs in both basal conditions and during CCCP induced mitochondrial depolarizaiton.

**Response authors:** We agree with the reviewer that it would be informative to interrogate the localization of Parkin in PBMCs in both basal conditions and during CCCP-induced mitochondrial depolarizaton. In the revised manuscript, we have included new immunocytochemical experiments with Jurkat cells and PBMCs that investigate if Parkin is recruited to depolarized mitochondria in these cell types (Fig. 7).

6. PLOS authors have the option to publish the peer review history of their article ([what does this mean?](https://eur01.safelinks.protection.outlook.com/?url=https%3A%2F%2Fjournals.plos.org%2Fplosone%2Fs%2Feditorial-and-peer-review-process%23loc-peer-review-history&data=02%7C01%7C%7C8290d351010d429a8e1a08d7bbdc6aa7%7C1faf88fea9984c5b93c9210a11d9a5c2%7C0%7C0%7C637184427667958977&sdata=TU5vFJvDTxhsf8UUeF9WQQiPeagIU7RhBAfKGq8ZuxM%3D&reserved=0)). If published, this will include your full peer review and any attached files.

If you choose “no”, your identity will remain anonymous but your review may still be made public.

**Do you want your identity to be public for this peer review?** For information about this choice, including consent withdrawal, please see our [Privacy Policy](https://eur01.safelinks.protection.outlook.com/?url=https%3A%2F%2Fwww.plos.org%2Fprivacy-policy&data=02%7C01%7C%7C8290d351010d429a8e1a08d7bbdc6aa7%7C1faf88fea9984c5b93c9210a11d9a5c2%7C0%7C0%7C637184427667968971&sdata=IWYpPxOVciVZrY3DBmFWeIImfAXclacJxPjoFJfQVow%3D&reserved=0).

Reviewer #1: No

Reviewer #2: No
[NOTE: If reviewer comments were submitted as an attachment file, they will be attached to this email and accessible via the submission site. Please log into your account, locate the manuscript record, and check for the action link "View Attachments". If this link does not appear, there are no attachment files to be viewed.]

While revising your submission, please upload your figure files to the Preflight Analysis and Conversion Engine (PACE) digital diagnostic tool, [https://pacev2.apexcovantage.com/](https://eur01.safelinks.protection.outlook.com/?url=https%3A%2F%2Fpacev2.apexcovantage.com%2F&data=02%7C01%7C%7C8290d351010d429a8e1a08d7bbdc6aa7%7C1faf88fea9984c5b93c9210a11d9a5c2%7C0%7C0%7C637184427667968971&sdata=O4qRm3GvkHvgTRBzxfKuS7nW1%2F2EjQ1gGtnmdgfD6ao%3D&reserved=0). PACE helps ensure that figures meet PLOS requirements. To use PACE, you must first register as a user. Registration is free. Then, login and navigate to the UPLOAD tab, where you will find detailed instructions on how to use the tool. If you encounter any issues or have any questions when using PACE, please email us at [figures@plos.org](mailto:figures@plos.org). Please note that Supporting Information files do not need this step.
